# Molecular Dynamics Simulation of Fe-Based Metal Powder Oxidation during Laser Powder Bed Fusion

**DOI:** 10.3390/ma15186394

**Published:** 2022-09-15

**Authors:** Yu Wang, Xianglin Zhou

**Affiliations:** State Key Laboratory for Advanced Metals and Materials, University of Science and Technology Beijing, Beijing 100083, China

**Keywords:** molecular dynamics simulation, laser powder bed fusion (L-PBF), reaction force field, oxidation

## Abstract

Because the laser powder bed fusion process is generally completed in a confined space and in a very short time, it is difficult to study material oxidation during this process using traditional methods. To address this knowledge gap, in this work, we used molecular dynamics (MDs) based on a reaction force field (ReaxFF) to clarify the atomic-level interaction mechanism between metal atoms and oxygen molecules during laser powder bed fusion. The ReaxFF potential energy model has variable charges that can dynamically handle charge changes between atoms and the breaking and formation of chemical bonds that occur during oxidation reactions. We investigated the effects of laser power, scanning speed, region position, and oxygen concentration on powder oxidation. The results show that the laser power and scanning speed affected the oxidation degree by changing the energy input density, and the oxidation degree increased with the energy input density. Different forms of oxidation occurred near the melt channel due to the existence of a temperature gradient, and the degree of oxidation increased with the temperature. Atoms in the metal powder model underwent selective oxidation, which was related to the potential energy of their atomic position. A larger potential energy made it easier for iron atoms to overcome the energy barrier during the initial stage of oxidation, making them easier to oxidize.

## 1. Introduction

Additive manufacturing (AM) is an advanced manufacturing technology that was developed in the 1980s. After three-dimensional modeling, the metallic material is radiated through a high-energy-density heat source to rapidly heat and solidify it. The component is gradually completed by points, lines, and surfaces [1,2]. In particular, the laser powder bed fusion (L-PBF) technique is suitable for obtaining complex metallic objects due to its geometric freedom in selective sintering of powders [3]. As a technology that subverts traditional casting methods, it has a high forming accuracy, short manufacturing process, and near-net molding. It is expected to change the manufacturing method of existing parts, accelerate the design of product parts, and realize customized rapid prototyping of final products [4,5]. L-PBF has been used to manufacture steel materials for components in the aerospace, biomedical, high-performance molds, and nuclear industries [6,7]. It has been successfully used to design and form steel materials such as M2 tool steel, 316L stainless steel, and porous structural stainless steel [8,9]. The nuclear industry exploits the unique capabilities of L-PBF in reactor design to directly prototype and manufacture parts for new designs, as well to rapidly manufacture replacements for obsolete parts during plant refueling outages, and to rapidly deploy custom-designed parts [10,11,12].

However, there are still great challenges preventing the large-scale applications of iron-based alloy L-PBF, particularly the large-scale production of parts or the in situ repair of metal parts. Since the purity of the noble gas cannot reach 100%, and the construction of the inert gas chamber for large components increases the cost of repair, it is difficult to use inert gas to prevent component oxidation during in situ repair. Experimental studies have shown that oxidation during L-PBF greatly affects the mechanical properties of components, such as their strength, hardness, and ductility [13,14,15]. Peter et al. used in situ X-ray diffraction to study the effect of metal oxidation on molten pool dynamics and defect formation during L-PBF. They found that metal oxides changed the direction of the metal fluid [16]. Simchi et al. found that metal oxides in the molten pool reduced the fluidity of the powder, resulting in a difference in the bulk density of the powder, which increased the porosity of the resulting components and generated cracks [17]. Gu et al. found that oxides reduced the wettability of the molten pool, led to balling, and increased the surface roughness of the resulting parts [18].

Current research on oxidation during L-PBF mainly uses scanning electron microscopy (SEM), energy-dispersive spectroscopy (EDS), and X-ray diffraction (XRD) to analyze the surface morphology, element distribution, and phase composition after oxidation. The research is more concerned about the influence of the original powder oxidation or component oxidation on the mechanical properties [19,20,21,22]. Lou et al. investigated the effect of oxide inclusions in laser additively manufactured stainless steel on impact toughness and stress corrosion cracking behavior [15]. Peter et al. investigated the effect of powder oxidation on defect formation in laser additive manufacturing [16]. There are few studies on the oxidation process. The reason for this phenomenon is that in L-PBF, the unmelted metal powder is exposed to high temperatures for a short time, usually 10^−6^–10^−3^ s [23], and it difficult to measure these oxides using traditional methods. It is especially difficult to precisely elucidate the interaction mechanism between metal surfaces and oxygen at the atomic level. Some scholars have used first-principles and molecular dynamics (MDs) simulations to study the oxidation of metals. Blonski et al. used first-principles calculations to study the dissociative adsorption of O_2_ molecules on clean and oxygen-precoated Fe surfaces [24]. Wu et al. used molecular dynamics to accurately calculate the physical properties of metals, such as their elastic constant [25], diffusivity [26], and melting point [27].

However, the first-principles method is limited to dealing with sufficiently small systems (fewer than 100 atoms), and their computational costs are much higher when combined with L-PBF dynamic simulations. The alternative molecular dynamics simulations using empirical force fields, while still being applicable to larger systems (up to 10,000 atoms), cannot handle chemical bond formation and breaking. Therefore, it cannot be used to describe a metal surface’s chemical reactions during L-PBF.

Because of the enormous practical importance of iron-based metallic materials, an in-depth understanding of Fe oxidation and oxide growth at the atomic level is important for the wide application of L-PBF. It will also provide basic knowledge for further studying the use of iron-based alloys to create more complex components. Therefore, this work’s main focus was to study the dynamic mechanism and influencing factors of iron oxide formation and growth during L-PBF at the atomic level by employing computational simulations.

## 2. Simulation Processes and Methods

Van Duin et al. proposed the reaction force field (ReaxFF) in 2001, which is a potential function based on quantum mechanical calculations and a powerful tool for developing and optimizing material chemistry. Its emergence built a bridge between quantum mechanical calculations and force field simulations [28,29]. Molecular dynamics simulation based on ReaxFF has been successfully applied to metal oxidation [30,31,32], which has great advantages when studying the mechanism of metal oxidation. They can provide a visual atomic map of the simulated system, allowing the analysis of oxidation processes. There is no need for preset reaction paths when conducting chemical reaction simulations. By obtaining the force field parameters from a quantum chemical calculation training set, the formation and dissociation of chemical bonds can be reproduced by using the continuous bond order and interatomic distance between atoms, which is very important for reaction calculations of complex systems [28].

### 2.1. ReaxFF Force Field

The ReaxFF utilizes the relationship between bond length, bond order, and bond energy to achieve a smooth transition between bonded and non-bonded systems. It can simulate the chemical bond formation and breaking phenomena that cannot be simulated by traditional force fields and is more suitable for describing chemical processes such as gas adsorption. Moreover, it has high computational efficiency and can simulate systems with thousands of atoms over millions of time steps [33].

The total energy of the system described by ReaxFF is expressed in Equation (1):

(1) $$E_{system}=E_{bond}+E_{val}+E_{tors}+E_{over}+E_{under}+E_{VdW}+E_{Coul}+E_{species}$$

where $E_{bond}\;$is the energy associated with forming atomic bonds;$\;E_{val}$ is the valence angle energy; $E_{tors}$ is torsion angle energy; $E_{over}$ and $E_{under}$ are the overcoordination and undercoordination penalty energies generated by calculations based on the valence rule, respectively; $E_{VdW}$ and $E_{Coul}$ are terms for handling non-bonding interactions, namely, van der Waals forces and Coulombic interactions. These energy terms are calculated under the reaction force field according to the bond order between atoms. The interatomic bond order is an algorithm for interatomic chemical bonds proposed by Abell [34], Tersoff [35], Brenner [36], and others when they studied covalent systems. The calculation formula of the bond order between two atoms, *i* and *j*, is $BO_{ij}$:

(2) $$BO_{ij}=BO_{ij}{}^{\alpha }+BO_{ij}{}^{\pi }+{BO_{ij}}^{\pi \pi }\;=exp\left[p_{bo1}{\left(\frac{r_{ij}}{r_{0}^{\sigma }}\right)}^{p_{bo2}}\right]+exp\left[p_{bo3}{\left(\frac{r_{ij}}{r_{0}^{\pi }}\right)}^{p_{bo4}}\right]+exp\left[p_{bo5}{\left(\frac{r_{ij}}{r_{0}^{\pi \pi }}\right)}^{p_{bo6}}\right]$$

In Equation (2), $r_{ij}$ is the distance between atoms *i* and *j*; $r_{0}$ is the bond length at equilibrium; $p_{bo}$ is an empirical parameter; $BO_{ij}$ mainly depends on the interatomic distance and the local chemical environment around the atoms.

Charge transfer between cations and anions is dynamically determined at each molecular dynamics step by applying the electronegativity equilibrium (EEM) and QEq method, which is calculated as follows:

(3) E(q)=∑i[χiqi+ηiqi2+Tap(rij)kcqiqj(rij2+rij−3)13]

where q is the ion charge; χ is electronegativity; η is the atomic hardness; Tap(r) is the seventh-order taper function; r and $k_{c}$ are the shielding parameter and dielectric constant, respectively. More comprehensive details on the ReaxFF can be found in the literature [28,29,37].

In this work, the ReaxFF parameters for Fe-O developed by Aryanpour were used to describe the reaction between Fe and O during L-PBF [32]. The complexity of changing interatomic bonding properties in the oxide and metal regions was addressed by considering dynamic charge transfer between different species. This force field has been applied to analyze the migration of oxygen atoms at the Fe/FeO interface, the oxidation of iron in supercritical water [33], iron oxidation under typical humid conditions [34,35], and the effect of oxidation on the deformation of iron nanowires [36].

### 2.2. Model Setup

**Figure 1:**
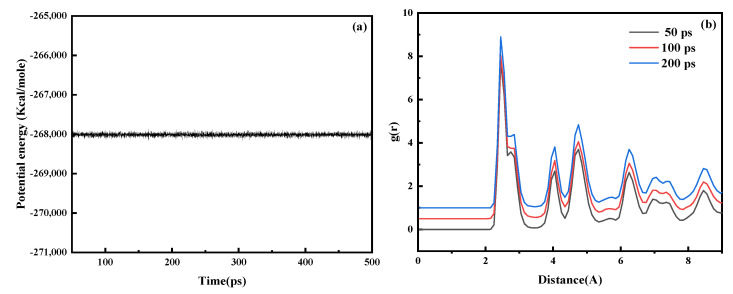
Effect of relaxation time on the model structure of iron nanoparticles. (**a**) Variation in the system’s potential energy with time. (**b**) Relaxation times of 50 ps, 100 ps, and 200 ps were used for the double−body distribution function of Fe−Fe.

**Figure 2:**
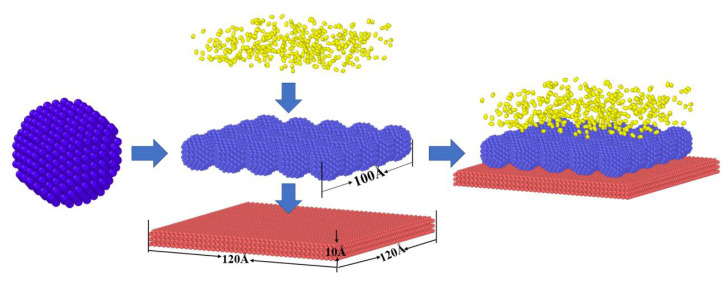
Molecular dynamics model of the L-PBF process. (Red represents substrate atoms, blue represents metal powder atomic particles, and yellow represents oxygen molecules.)

The model established to explore the formation of iron oxides in the L-PBF process consisted of three parts: spherical metal powder, iron substrate, and oxygen molecules randomly distributed over the metal powder. The model was established in three steps. In the first step, an iron nanoparticle model with a radius *r* = 10 Å was established using LAMMPS [38,39] as powder particles. The second step involved using the conjugate gradient method to minimize the energy of the established iron nanoparticle model, which was relaxed at 300 K for 200 ps to obtain the surface structure with the lowest energy. The relaxation results are shown in Figure 1a,b. The system’s potential energy and Fe-Fe binary distribution function change with time demonstrated that a relaxation time of 200 ps was sufficient to stabilize the iron nanoparticle structure. The third step replicated the obtained iron nanoparticles 5 × 5 × 1 times in three directions. Finally, these powders were placed on a 120 Å × 120 Å × 10 Å iron substrate. When exploring the oxidation of iron atoms, to save computing resources and accelerate the reaction rate, a certain number of oxygen molecules were randomly distributed in the space 30 Å above the iron atoms. The established model is shown in Figure 2. Table 1 lists the structural details of the corresponding models.

### 2.3. Simulation Parameters

The initial velocity was set to the velocity of atoms at 300 K. The velocity of the atoms was assigned to each atom through a Maxwell–Boltzmann distribution according to the temperature generated by laser scanning, which conformed to the laws of statistical physics. The model was equilibrated at 300 K using an NVT ensemble before loading the laser light source and then further relaxed using an NVE ensemble so that the whole simulation system reached equilibrium at room temperature. During the relaxation process, the charge of each atom was calculated by the charge equilibration (QEq) method. Since the charge relaxation algorithm used to minimize electrostatic energy is very time-consuming, the atomic charge was updated every 10 time steps. This method improves the calculation efficiency and ensures the accuracy of the results, so it is more commonly used.

### 2.4. Loading of the Laser Light Source

**Figure 3:**
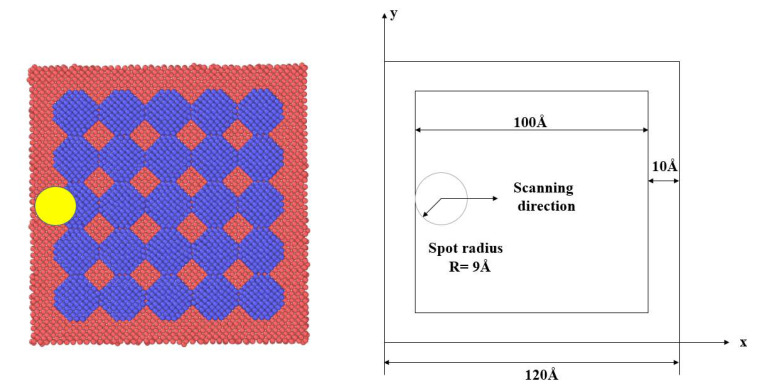
Initial position and the scanning direction of the laser during simulation. (Red represents substrate atoms, blue represents metal powder atomic particle.)

Lasers used in L-PBF technology are generally long-pulse continuous lasers, such as Nd: YAG lasers or fiber lasers. Long-pulse continuous lasers and ultra-short-pulse lasers interact with metallic materials via completely different mechanisms. The local temperature during the interaction with metallic materials is relatively low, which hardly induces the absorption of photons by electrons. Therefore, when studying the L-PBF process, we can only consider the effect of the heat generated by the laser beam on the model while ignoring the effects of electrons and phonons. In this simulation, the laser beam was loaded using the “fix heat” command in LAMMPS, which simulated the laser heating process by adding non-translational kinetic energy to the surrounding atoms through the laser spot. We assumed that the laser energy density conformed to a Gaussian distribution. The initial position and the scanning direction of the laser beam are shown in Figure 3. The center position of the laser light source was (10, 50).

### 2.5. Simulation of Laser Powder Bed Fusion Process

The process for simulating L-PBF is divided into two stages: heating and cooling. During the heating process, the “fix heat” command was used to load the laser beam, and the laser beam was scanned in a single channel along the *x* direction at a fixed speed. During laser scanning, the iron substrate was kept at 300 K by the Langevin temperature method. After scanning, the laser beam was removed from the system, and the substrate temperature was controlled at 300 K with the Langevin method to further cool the powder. During the laser scanning melting process, periodic boundary conditions were used in the *x* and *y* directions, while shrink boundary conditions were used in the *z* direction. The time step used for the whole scanning process was 1 fs. In order to save computing resources, the actual process parameters are scaled to establish the simulation parameters used in this paper. Therefore, the process parameters used in this paper are quite different from the parameters in the actual L-PBF process. However, the simulation results of previous work show that this scaling has practical significance and can still provide a trend consistent with the experimental results [40,41,42]. The parameters involved in the L-PBF simulation process are shown in Table 2.

## 3. Results and Discussion

### 3.1. The Effect of Process Parameters on The Degree of Oxidation

For L-PBF, the main parameters were laser power, spot radius, and scanning speed [43,44,45]. To explain the influence of parameters on the oxidation degree from an atomic perspective, the simulation calculation of the L-PBF process was performed by changing parameters including the power, scanning speed, and spot diameter. The specific process parameters are shown in Table 2. When iron is oxidized, oxidation products with different valence states are often generated. In order to express the oxidation process more conveniently, the oxygen atoms in the scanning area are screened by the charge screening function. The number of oxygen atoms with a charge of −1 was counted to indicate the degree of oxidation.

**Figure 4:**
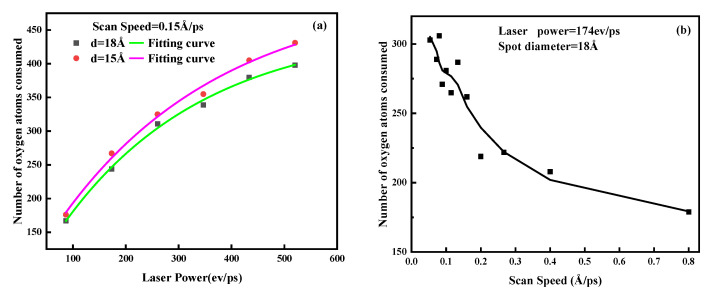
The effect of processing parameters on the degree of oxidation during scanning: (**a**) laser power and (**b**) scanning speed.

To investigate the effect of power input on powder oxidation during L-PBF, single fusion lines were generated by scanning metal powders at laser powers ranging from 100 to 500 eV/ps using laser beams with spot radii of 15 Å and 18 Å. For a given scan parameter, the effect of laser power on the degree of oxidation is exponential. Figure 4a shows the effect of power on the degree of oxidation under different spot diameters. Upon increasing the power, the energy transmitted by the laser to the powder increased, the metal atoms absorbed more energy, and the degree of oxidation increased. When the laser power was constant, a larger laser spot radius resulted in a lower oxidation degree of the metal atom. Figure 4b shows the effect of scanning speed on the oxidation degree. Upon continuously increasing the scanning speed, the oxidation degree of the metal atoms decreased significantly.

The effect of laser power and scanning speed on the L-PBF process can be attributed to energy density [43,46,47], $E_{d}$, which represents the energy transferred to a certain point and is calculated using the following formula:

(4) $$E_{d}=\frac{P}{V_{S}\cdot h\cdot d}$$

where $E_{d}$ is the energy density, *P* is the laser power, $V_{S}$ is the laser scanning speed, *h* is the distance between the melt channels, and *d* is the thickness of the powder layer [48]. In this work, due to the use of single-layer single-channel scanning, the parameters that determined the laser energy density only included the laser power *P* and the scanning speed $V_{S.}$ Increasing the laser power may have caused the temperature of the molten pool to rise. The extent to which the temperature of the melt pool increased depended on the energy accumulated by the difference between the rate of energy input and the rate of energy dissipation throughout the substrate. Therefore, the higher the laser power, the higher the energy density input, and the higher the molten pool temperature. The solubility of oxygen molecules in the molten pool increased, the oxidation reaction rate increased, and the oxidation degree increased. The influence of the laser spot size on the scanning process can be determined by the following formula:

(5) $$E_{d}=\frac{P}{V_{S}\cdot \pi r^{2}}$$

where $E_{s}$ is energy input per unit area, P is laser power, *r* is the spot radius, and $V_{S}$ is the laser scanning speed. According to Equation (5), when the laser power and scanning speed are the same, the larger the spot size, the smaller the energy input per unit area, the lower the solubility of oxygen molecules in the molten pool and the lower the oxidation reaction rate, and the degree of oxidation is reduced.

The effect of scanning speed on the L-PBF process is essentially determined by the duration of laser action in the scanning area. At relatively low scanning speeds, the laser–powder interaction time is long enough to allow the powder material to absorb energy, thereby expanding the molten area [49]. Due to an increase in temperature and the expansion of the molten area, there was a greater probability of oxygen molecules interacting with metal atoms. Therefore, a lower scanning speed resulted in a greater oxidation degree in the metal powder. However, this does not mean that a higher scanning speed will produce a component with better performance. As the scanning speed increased, the interaction time between the laser and the powder became shorter, and the size of the molten pool decreased. However, the diffusion speed of the molten pool was constant. When the scanning speed exceeds the diffusion speed of the molten pool, fusion defects such as powder entrainments may appear in a component due to incomplete melting of the metal powder, thereby degrading the mechanical properties of the component.

### 3.2. Oxidation Concentration near the Laser Melt Channel

**Figure 5:**
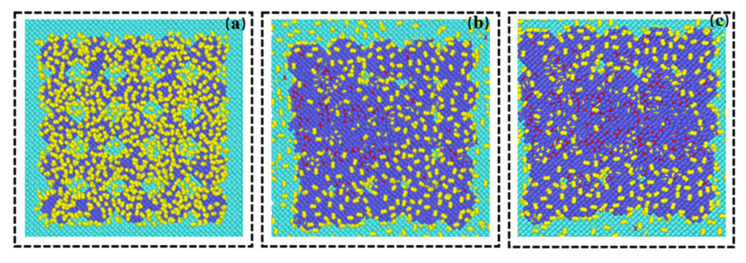
The arrangement of atoms during laser scanning (red atoms represent oxidized atoms) (**a**) before scanning, (**b**) during scanning, and (**c**) after scanning.

**Figure 6:**
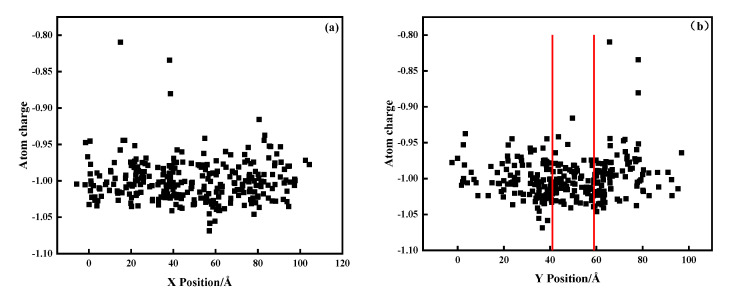
Relationship between oxidation sites and coordinates: (**a**) relationship with the *x* coordinate and (**b**) relationship with the *y* coordinate (the area between the two red lines represents the laser melting track.).

The trajectory files output by the LAMMPS calculation were post-processed and analyzed by Ovito. Figure 5a–c, respectively represent the arrangement state of each atom in the area before, during, and after laser scanning. Red atoms indicate oxidation. It can be observed that the oxidation of metal atoms was concentrated in the middle area. We believe that this oxidation phenomenon was related to the laser scanning weld. To further verify the connection between the oxidation and the laser scanning melt channel, we formed a relationship between the position of the oxidation atom and the *x* and *y* coordinates, as shown in Figure 6a,b.

**Figure 7:**
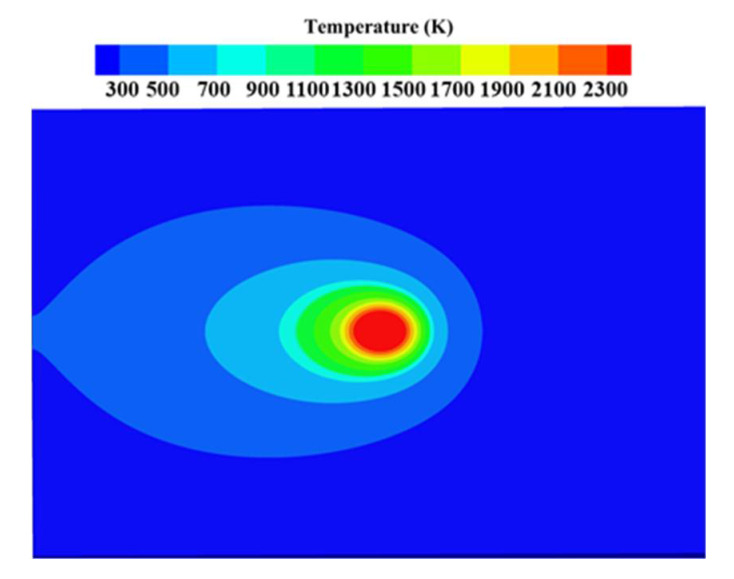
Temperatures in different areas within the molten pool.

From Figure 6a, we can observe that the distribution of oxidation positions along the *x* direction was relatively uniform, and oxidation occurred along the laser scanning path (0, 100). In Figure 6b, the location of the laser melt path is marked with a red line. Oxidation concentrated at (41, 59) was located near the melt channel. This phenomenon has also been reported in ref. [45]. During the simulation, when the incident laser beam scanned the metal surface, most energy was absorbed by the powder particles, causing localized melting and forming a molten metal pool [44,46]. To study the phenomenon of oxidation concentration, the molten pool generated during the laser scanning process was simulated by finite element software. The simulation results are shown in Figure 7. There was a temperature gradient near the molten pool, which covered a range beyond the diameter of the laser spot. This phenomenon occurred due to thermal radiation from the powder layer and thermal conduction between powder particles. Therefore, the laser source that supplied energy to the powder layer not only generated a molten pool but also conducted heat near the molten pool. The area near the melt channel is called the heat-affected zone, and the oxidation concentration shown by the simulation results occurred in this area.

**Figure 8:**
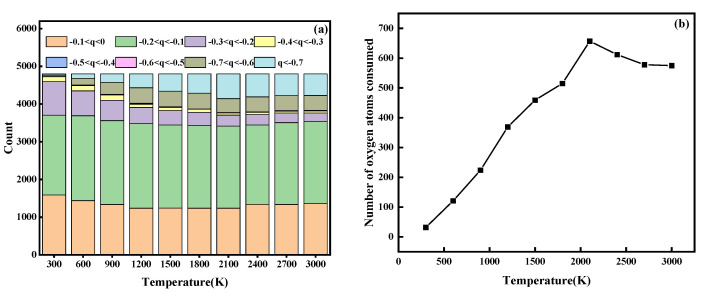
(**a**) The number of atoms with different charges at each temperature. (**b**) The effect of different temperatures on the degree of oxidation.

To further investigate the relationship between temperature and oxidation degree during L-PBF, we simulated powder oxidation at different temperatures, and the simulation results are shown in Figure 8. We found that the oxidation degree first increased and then decreased with the temperature, which confirmed that the oxidation concentration phenomenon near the melting channel was related to the temperature. We believe that the degree of preoxidation increased with the temperature because a higher temperature resulted in greater oxygen solubility in the molten metal. This increased the probability that oxygen molecules would react with the metal. The subsequent reduction in the degree of oxidation at higher temperatures may have been due to the evaporation of molten metal, which resulted in droplet splashing that caused the metal atoms to leave the oxidation statistical region.

### 3.3. Effect of Oxygen Concentration on the Oxidation Degree

**Figure 9:**
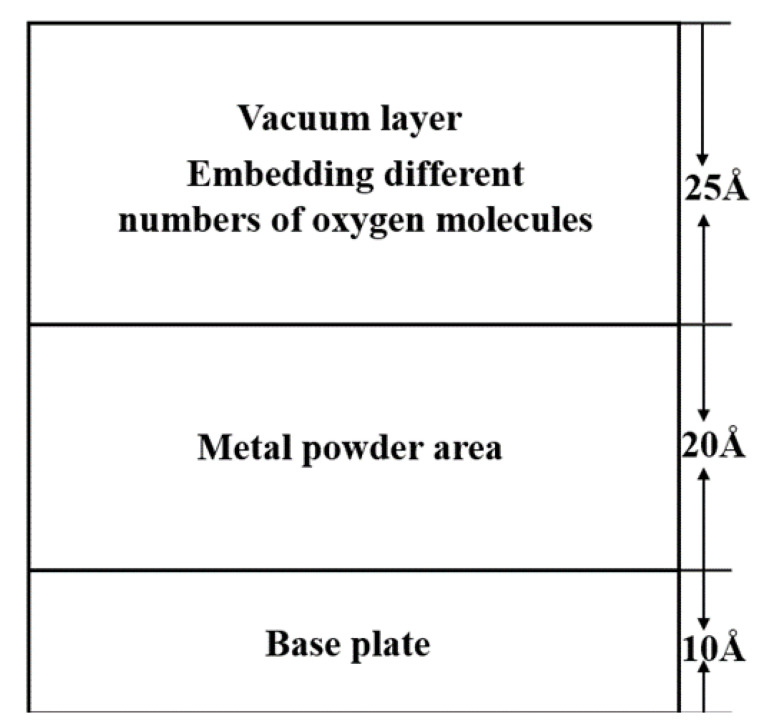
Schematic diagram for model of the effect of oxygen concentration on oxidation degree.

To explore the effect of oxygen concentrations on the degree of oxidation in the L-PBF, as shown in Figure 9, we established an oxygen consumption model. The oxidation of metal powders during L-PBF was simulated after embedding 400 to 5000 different numbers of oxygen molecules.

The calculation of oxygen consumption can be determined by the following formula:

(6) $$O_{consume}=O_{start}-O_{surplus}$$

**Figure 10:**
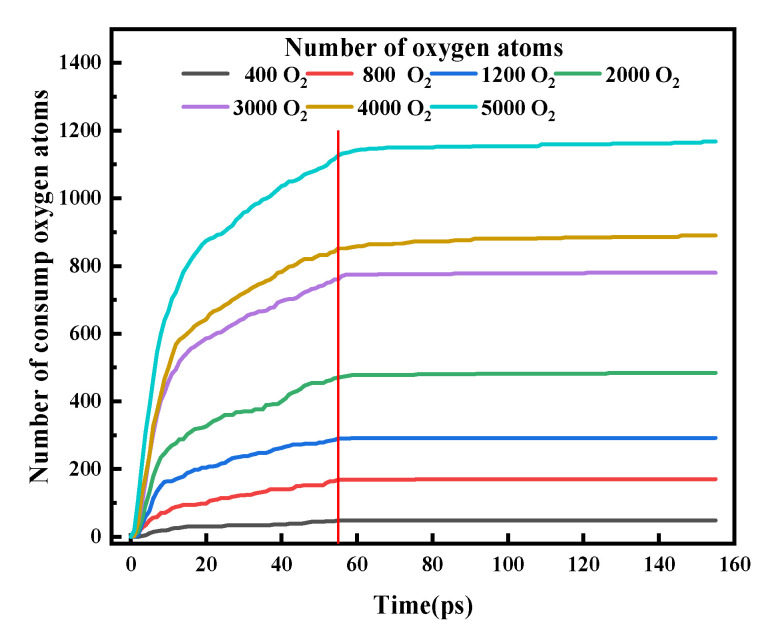
Oxygen consumption at different oxygen concentrations.

After the scan, the number of oxygen atoms with a charge of 0 should be counted, which is $O_{surplus}$. Figure 10 shows the amount of oxygen consumed with time at different oxygen concentrations, where 0–55 ps was the laser scanning stage, and after 55 ps was the cooling stage. Oxidation mainly occurred during the laser scanning stage, and almost no oxidation occurred during the cooling stage.

The oxidation that occurred during the 0–55 ps laser scanning process can be divided into two stages. In the first stage of oxidation, the curve slope was high, the consumption rate of oxygen molecules was high, and the oxidation speed was fast. In the second stage of oxidation, the curve tended to remain flat, the slope was smaller, the oxygen molecule consumption rate was lower, and the oxidation rate was slower. This is consistent with what was observed in the experiments of Vink and Campo et al. [50,51]. These two stages are described as the rapid oxidation stage and oxide growth stage in Wagner’s metal oxidation theory. During the oxidation stage, a single atomic layer of oxide rapidly formed on the metal surface for a very short period and then entered the oxide growth stage. The slowdown in the oxide growth stage was due to the outward diffusion of Fe atoms through the growing oxide film and the inward diffusion of O in the opposite direction. This process was limited by some basic physical and chemical steps, such as ion diffusion, electron transport, and electron transfer [52,53].

**Figure 11:**
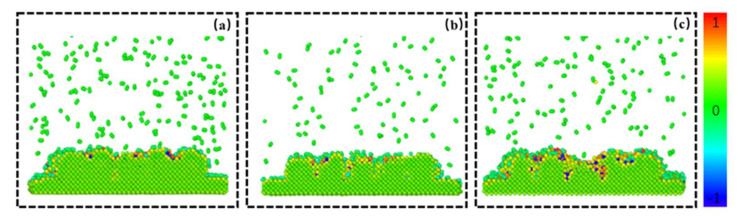
Cross-section of powder oxidation degree as a function of time at 2000 O_2_. Degree of powder oxidation at (**a**) 100 ps, (**b**) 300 ps, and (**c**) 500 ps.

Figure 11 reveals the variation in the oxygen atom diffusion depth with time. Over time, more oxygen atoms on the FeO/Fe layer diffused into deeper atomic layers and oxidized the internal metal atoms. The simulation results confirm that oxygen migrated from the oxide layer to the oxide–metal interface during the oxide growth stage, which is in good agreement with the results of Bachhav et al. [54].

When the laser scanning is completed, the coordinates of all oxygen atoms diffused into the metal powder at the laser center position (*y* = 50) are counted by Ovito, and the coordinates of the oxygen atoms in the depth direction (coordinate values in the z direction) are used to represent the thickness of the oxide film at each position. The average oxide film thickness, *d*, is calculated as follows:

(7) $$d=\frac{1}{n}\overset{n}{\underset{t=1}{\sum }}Z_{\left(t\right)}$$

where *d* is the average thickness of the oxide film, *n* is the number of oxygen atoms diffused into the metal powder, and $Z_{\left(t\right)}$ is the coordinate value of the oxygen atoms in the *z direction*. The growth of the oxide film at the center of the laser under different oxygen concentrations is shown in Table 3. The average oxide film thickness increased with the increase in oxygen concentration, but the thickness of each part of the oxide film was not the same under the same conditions. This shows that the diffusion of oxygen atoms in the metal powder was different, and the degree of oxidation of each part was also different. According to Fick’s law of diffusion, oxidation during the L-PBF is an unsteady diffusion. We believe that the difference in the diffusion of oxygen atoms was related to the potential energy at the location of the nearby iron atoms.

**Figure 12:**
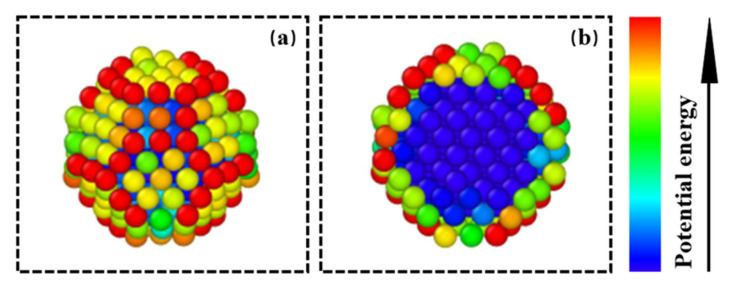
Potential energy of iron atoms at different positions within spherical particles. (**a**) The potential energy of each atom on the surface of the spherical particle; (**b**) the potential energy of each atom on the cross-section of the spherical particle.

We calculated the potential energy of iron atoms at various positions in the powder particle model. As shown in Figure 12, to represent the calculation results more intuitively, we colored the atoms differently according to the potential energy of the iron atoms at different positions. The results show that surface atoms, especially those located at the corner and edge sites, had higher potential energies, while the potential energy of the atoms located inside the spherical particles was lower. We believe this difference was related to the coordination number. Atoms located at the surface and edges had a higher potential energy and reactivity due to lower coordination numbers. Therefore, oxygen molecules near these special positions more easily obtained energy, which made it easier to break through the energy barrier generated by the transfer of electrons from the metal to oxygen during the early stage of oxidation.

### 3.4. Oxidation Kinetics

Oxidation during L-PBF is a gas–liquid metal reaction whose oxidation mechanism involves chemical adsorption between the surface of a sample and the surrounding atmosphere [55]. In a high-temperature atmosphere, oxygen molecules in the vacuum chamber will collide with the surface of the sample and decompose into individual oxygen atoms. Therefore, chemisorption occurs via interactions between the free electrons of the alloy and oxygen atoms.

**Figure 13:**
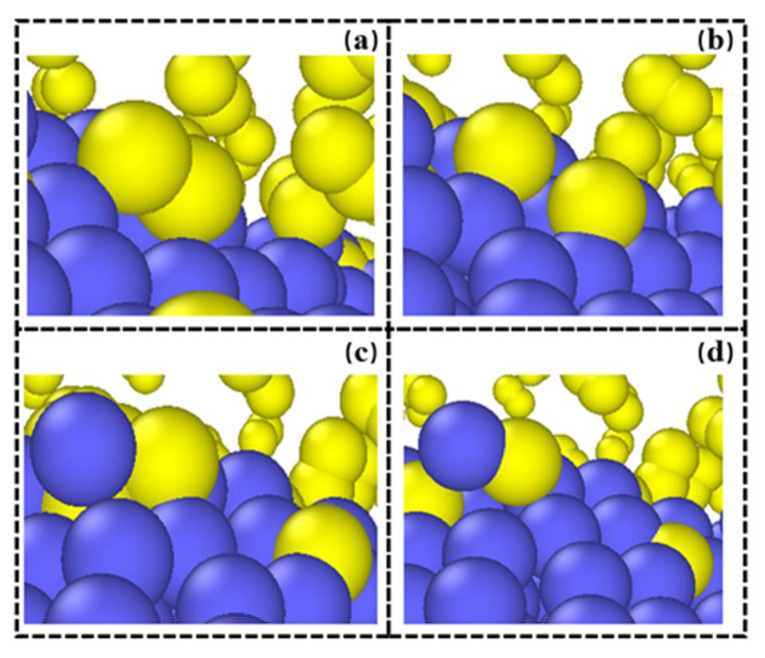
Oxygen molecule dissociation and metal atom oxidation process at (**a**) 100 ps, (**b**) 300 ps, (**c**) 500 ps and (**d**) 700ps.

Figure 13 shows the adsorption and dissociation process of oxygen molecules during laser scanning. From an atomic perspective, the dissociation of oxygen molecules from the metal surface during the L-PBF process can be divided into two stages. In the first stage, oxygen molecules are rapidly adsorbed by the free surface of metal atoms, and then dissociated or partially ionized. The dissociated oxygen atoms reconstruct the surface by exchanging positions with metal atoms. In the second stage, Fe is activated after forming a chemical bond with O. Because of this, interactions with other metal atoms on the surface are weakened, allowing Fe atoms to potentially leave the surface.

**Figure 14:**
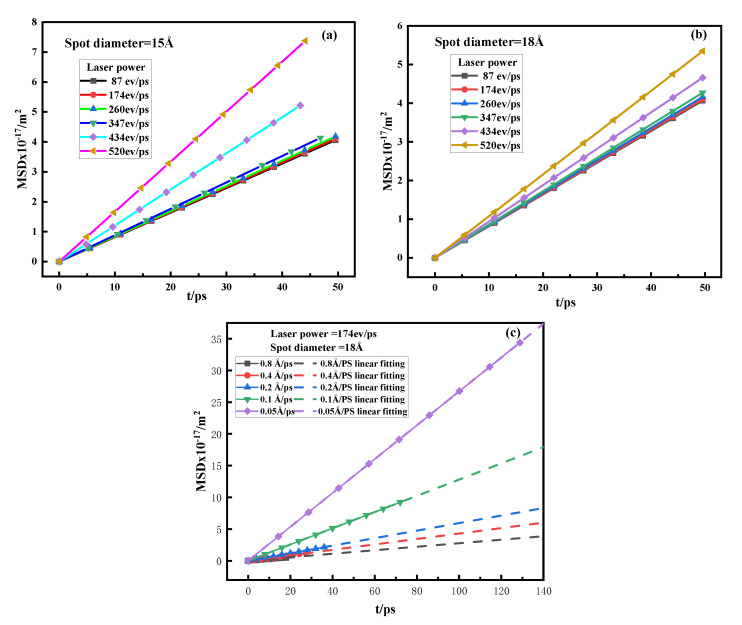
MSD curve of different process parameters. (**a**) MSD curves of different powers when the spot diameter is 15 Å. (**b**) MSD curves of different powers when the spot diameter is 18 Å. (**c**) MSD curves at different scan speeds.

The metal oxidation process mainly includes the adsorption of oxygen molecules on the metal matrix, the dissociation of the matrix surface, and the diffusion of oxygen or matrix atoms. Among them, the diffusion process dominates the oxidation kinetics. Studies have shown that when iron-based metals are oxidized, the diffusion rate of oxygen atoms is much greater than the diffusion rate of iron atoms [56]. Therefore, the growth of the oxide film is mainly dominated by the insertion and diffusion of oxygen atoms in the matrix. The diffusion coefficient of a substance is a physical property of a substance, which can express its diffusivity. Although LAMMPS cannot directly calculate the diffusion coefficient, it is known from the Einstein diffusion relationship that the diffusion coefficient can be obtained by calculating the mean square displacement (MSD) of atoms [57]:

(8) $$D=\frac{1}{6N}\underset{t\to \infty }{\lim}\frac{d}{dt}\overset{N}{\underset{i=1}{\sum }}{\left[r_{i}\left(t_{i}\right)-r_{i}\left(t_{0}\right)\right]}^{2}\approx \frac{1}{6}\frac{\partial }{\partial t}\left(MSD\right)$$

where D is the diffusion coefficient of the system, N is the total number of particles, t is the time, and $r_{i}\left(t_{i}\right)$ and $r_{i}\left(t_{0}\right)$ are the positions of the particles at $t_{i}$ and $t_{0}$, respectively. From Equation (8), the diffusion coefficient can be approximated as the ratio of the mean square displacement (MSD) to time. At a certain temperature, the diffusion coefficient is 1/6 times the slope of the atomic MSD curve. According to the results of oxidative diffusion, we made the MSD curve related to this paper, and the obtained results are shown in Figure 14a–c, which are the MSD curves under different spot diameters, different laser powers, and different scanning speeds.

In Figure 14a–c, the MSD curves under different conditions are basically straight lines. The slope of the straight line was fitted by MATLAB, and the diffusion coefficient of each element was calculated by substituting Equation (8). The calculation results are shown in Table 4 and Table 5.

As seen in Table 4 and Table 5, the laser power, spot diameter, and scanning speed all had an effect on the diffusion coefficient of oxygen atoms in the metal powder. Process parameters have the same effect on the diffusion coefficient as they do on the degree of oxidation. The reason for this phenomenon is that the size of the diffusion coefficient is related to the temperature [58]:

(9) $$D=D_{0}\cdot \exp\left(\frac{-Q}{RT}\right)$$

where *R* is the gas constant, and $D_{0}$ and Q are the diffusion constant and activation energy, which are related to the properties of the material. From Equation (9), it can be seen that the diffusion coefficient is only related to temperature. Therefore, in the process of L-PBF, the essence of the effect of process parameters on the degree of oxidation is to affect the temperature by changing the input of energy density, thereby affecting the diffusion coefficient. The higher the input energy per unit time, the higher the temperature in the molten pool area, the greater the diffusion coefficient of oxygen atoms, and the greater the final oxidation degree of the metal.

## 4. Conclusions

In this work, we performed reactive molecular dynamics simulations of the L-PBF to study the oxidation of metal powders, and came to the following conclusions:

(1) The L-PBF parameters had a great influence on the degree of metal oxidation. The greater the laser power, the greater the degree of metal oxidation, the greater the scanning speed, and the smaller the degree of metal oxidation. We summarized the influence of parameters in terms of energy density. The laser power and scanning speed affected the input energy density; the greater the energy input per unit time, the higher the temperature of the resulting melt pool, and the larger the melt pool area. The greater the probability of interactions between oxygen molecules and metal atoms on the surface of the molten pool, the greater the degree of metal oxidation.
(2) Due to the existence of thermal radiation and thermal conduction between metal atoms, the energy input by the laser caused a temperature gradient in the molten pool and nearby areas. The temperature distribution area was divided into the melting zone and heat-affected zone. In the heat-affected zone, we observed an oxidation con-centration phenomenon that was related to temperature. The solubility of oxygen molecules in the molten metal increased with the temperature, which increased the probability of contact and dissociation between metal atoms and oxygen molecules, thus increasing the oxidation degree of metal.
(3) When studying the effect of oxygen concentration on the oxidation degree, the simulation process was divided into two parts: scanning and cooling. Oxidation mainly occurred during laser scanning and hardly occurred during cooling. The average thickness of the oxide film increased as the oxygen concentration increases. Oxygen atoms diffused in the metal matrix in an unsteady state. Under the same oxygen concentration, the thickness of each part of the oxide film was not the same. The thickness of the oxide film is related to the potential energy of the iron atoms in the region. The atoms located at the edges and corners had higher atomic potential due to their low coordination number, and it was easier to break through the energy barrier generated by the transfer of electrons from metal to oxygen in the early stage of oxidation, so the oxide film near this position was thicker.
(4) In the L-PBF process, the oxidation mainly includes the adsorption of oxygen molecules on the metal matrix, the dissociation of the matrix surface, and the diffusion of oxygen or matrix atoms. The diffusion rate of oxygen atoms is much higher than that of iron atoms, the growth of oxide films is mainly dominated by the insertion and diffusion of oxygen atoms in the matrix. By calculating the diffusion coefficient of oxygen atoms under different process parameters, we found that the effect of process parameters on the diffusion coefficient was consistent with the effect on the degree of oxidation. The degree of metal oxidation increased with the increase in the diffusion coefficient. This proves that in L-PBF, the degree of oxidation of the building blocks is mainly controlled by diffusion. Process parameters affect the temperature by affecting the energy input. The higher the temperature, the greater the diffusion coefficient of oxygen atoms in the metal and the greater the degree of oxidation.

## Figures and Tables

**Table 1:** Details of the model created for the simulation.

Structure Name	Number of Atoms	Cell Size/Å
Single ball	339	*R* = 10
Power	8475	100 × 100 × 20
Base	10335	120 × 120 × 10
Oxygen	3862	100 × 100 × 30

**Table 2:** Parameters used in various parts of the simulation process.

	Laser Power (ev/ps)	Spot Diameter (Å)	Scan Speed (Å/ps)
The effect of laser power on the degree of oxidation in Section 3.1.	87	15 and 18	0.15
	174		
	260		
	347		
	434		
	520		
The effect of scanning speed on the degree of oxidation in Section 3.1.	174	18	0.8
			0.4
			0.2
			0.16
			0.10
			0.08
			0.05
Others	174	18	0.16
Number of oxygen atoms	Except for 3.3, the number of oxygen atoms is 2000.		

**Table 3:** Growth of oxide film at center of laser under different oxygen concentrations.

Number of Oxygen Molecules	Maximum Depth of Oxygen Atom/Å	Minimum Depth of Oxygen Atom/Å	Average Thickness of Oxide Film/Å
400	2.8351	0.943	1.227
800	3.136	0.667	1.291
1200	4.909	0.796	1.940
2000	5.207	0.761	2.066
3000	5.724	0.654	2.375
4000	6.290	0.362	2.486
5000	7.653	0.484	3.012

**Table 4:** Diffusion coefficients of oxygen atoms at different powers ($D\times 10^{-9}/m^{2}\cdot s^{-1}$).

Laser Power (ev/ps)	87	174	260	347	434	520
Spot diameter =15 Å	1.366	1.381	1.407	1.471	2.011	2.785
Spot diameter =18 Å	1.368	1.380	1.401	1.437	1.569	1.799

**Table 5:** Diffusion coefficients of oxygen atoms at different scan speeds ($D\times 10^{-9}/m^{2}\cdot s^{-1}$).

Scan Speed (Å/ps)	0.8	0.4	0.2	0.1	0.05
$D\times 10^{-9}/m^{2}\cdot s^{-1}$	0.4598	0.7147	0.9890	2.1317	4.4492
